# Perclose ProGlide closure devices vs. surgical removal for veno-arterial extracorporeal membrane oxygenation decannulation: a meta-analysis

**DOI:** 10.3389/fcvm.2025.1482305

**Published:** 2025-02-28

**Authors:** Qian Zhang, Ke Guo, Yunping Liu, Wei Wei, Kan Zhao, Haijun Huang, Zuoyi Yao

**Affiliations:** Department of Vascular Surgery, Chengdu Fifth People’s Hospital, Chengdu, Sichuan, China

**Keywords:** ECMO, surgical removal, perclose ProGlide, pre-closure, post-closure

## Abstract

**Objective:**

Perclose ProGlide closure devices (PPCDs) have become a more commonly used strategy in veno-arterial extracorporeal membrane oxygenation (VA-ECMO) decannulation, but there is still uncertainty regarding their efficacy and safety compared to surgical removal (SR). Therefore, we conducted a meta-analysis to compare the application results of the two methods in VA-ECMO decannulation.

**Methods:**

Data from PubMed, Cochrane Library, and EMBASE databases were systematically searched through May 2024. Prospective or retrospective studies on the comparison of PPCDs and SR in VA-ECMO decannulation were included. The outcomes included technical success, bleeding events, infections at the decannulation site, vascular complications, overall complications, mortality and duration of hospitalisation.

**Results:**

Eight retrospective studies involving 618 patients were included. The results demonstrated that PPCDs significantly reduced infections at the decannulation site and overall complications [odds ratio (OR) = 0.14, 95% confidence interval (CI) 0.05–0.44, *P* < 0.001], (OR = 0.27, 95% CI 0.16–0.48, *P* < 0.001). No significant differences were observed in the incidence rates of bleeding events, vascular complications, mortality and duration of hospitalisation between the two groups (*P* > 0.05). Subgroup analysis revealed that the SR group had a significantly higher risk of the removal site infection compared to the percutaneous pre-closure group (OR = 0.06, 95% CI 0.01–0.29, *P* = 0.0003).

**Conclusion:**

Pre-closure techniques utilizing PPCDs demonstrate a significant advantage over SR in reducing the overall incidence of complications for VA-ECMO decannulation, particularly in terms of reducing infections at the decannulation site.

## 1 Introduction

Veno-arterial extracorporeal membrane oxygenation (VA-ECMO) has been extensively employed as a temporary mechanical circulatory support system for patients with refractory cardiogenic shock, cardiac arrest, and high-risk cardiac intervention (1). The size of the deployed arterial cannula typically ranges from 15Fr to 21Fr to ensure sufficient perfusion. Given the size and accessibility of blood vessels, the femoral artery is the most commonly chosen. While manual pressure suffices for hemostasis after venous cannula removal, arterial decannulation necessitates a specific vascular repair technique. The traditional standard strategy for VA-ECMO arterial decannulation is surgical removal (SR), but it often entails risks such as inguinal hematoma, infection or delayed healing at the incision site, and damage to lymphatic or neural structures (2).

In recent years, many medical centers have adopted percutaneous vascular closure devices as the preferred strategy for femoral artery cannula removal in VA-ECMO, including the suture-based Perclose ProGlide closure device (PPCD, Abbott Vascular, Abbott Park, Illinois) and the plug-based MANTA closure device (MCD, Teleflex Inc., Wayne, Pennsylvania) (3–8). A recent meta-analysis has demonstrated that both PPCDs and MCDs are equally safe and efficacious for VA-ECMO decannulation, while PPCDs are associated with higher device failure (9). However, even if the initial hemostasis is unsuccessful during the decannulation process with PPCDs, it can be achieved by inserting a new closure device through a guide wire. For the MCDs, the inability to reinsert a guide wire precludes a secondary attempt at hemostasis. If hemostasis remains unattainable, prompt surgical intervention becomes mandatory. This contrast indicates that PPCDs have a broader applicability in clinical practice (10).

Currently, there are two methods for the application of PPCDs in VA-ECMO decannulation, including pre-closure techniques and post-closure techniques. The pre-closure technique refers to the percutaneous placement of non-absorbable sutures on the superficial wall of the femoral artery prior to inserting the large-bore sheath introducer. Hemostasis is achieved by tightening these sutures after removing the arterial cannula (11). Conversely, the post-closure technique involves inserting a guidewire and closure device through a puncture needle after femoral artery cannulation to achieve successful decannulation (12).

The studies by Unoki et al. and Hwang et al. indicated that there are no statistically significant differences in the incidence of adverse events between the use of PPCDs and SR in VA-ECMO decannulation (13, 14). In contrast, studies by Roberts et al. and Pellenc et al. observed a significantly reduced incidence of groin wound complications in patients who underwent decannulation using PPCDs (15, 16). The discordance among these study outcomes has the potential to perplex physicians in their clinical decision-making, thus necessitating timely summarization and analysis. Therefore, we conducted a comprehensive meta-analysis of all relevant publications on the use of PPCDs and SR for VA-ECMO decannulation. Our aim was to compare the efficacy and safety of these two methods and to investigate whether there are any significant differences in outcomes between the pre-closure and post-closure techniques when contrasted with SR.

## 2 Methods

### 2.1 Search strategy and selection criteria

This systematic review and meta-analysis was conducted in accordance with the Preferred Reporting Items for Systematic Reviews and Meta-Analyses (PRISMA) statement and was also registered at International Prospective Register of Systematic Reviews (http://www.crd.york.ac.uk/PROSPERO*;* identifier CRD42024545671) (17).

We conducted a search for pertinent literature up to May 2024 using the PubMed, Cochrane Library, and EMBASE databases. The first search term encompassed “extracorporeal membrane oxygenation,” “extracorporeal life support system,” and “mechanical circulatory support.” The second search term primarily targeted “Prolide,” encompassing related terms like “Perclose,” “ProStyle,” and “percutaneous.” The third search term pertained to “surgery,” encompassing terms such as “surgical,” “repair,” and “operation.” Supplementary Table S1 presents the detailed retrieval strategies that were employed in the PubMed, Embase, and Cochrane databases. Inclusion criteria were: (1) prospective or retrospective studies (randomized controlled trials or observational studies); (2) studies about VA-ECMO, with intervention methods of PPCDs or SR; (3) studies involving human subjects published in English. Exclusion criteria were: (1) studies with incomplete patient data, questionable laboratory data, and those without clear and quantitative results; (2) case reports, editorials or conference abstracts. A manual review of references and other relevant articles included in the study was conducted to minimize the risk of underreporting.

### 2.2 Study selection and data extraction

Two researchers independently assessed the eligibility of the studies based on their titles and abstracts. Disagreements between the researchers were resolved by a third researcher. Studies that met the inclusion criteria were selected for full-text evaluation. The relevant information was extracted and recorded for each selected study: first author, year of publication, country, total number of patients, study type and population characteristics. The primary outcome of interest was technical success rate. Secondary outcomes included infections at the decannulation site, bleeding events, vascular complications, overall complications, mortality and duration of hospitalisation.

### 2.3 Risk of bias and GRADE assessment

Two researchers independently used Newcastle-Ottawa scale (NOS) based on the selection of subjects, comparability between groups, and the assessment of exposure or outcome to evaluate and discuss literature quality until consistent scoring results were obtained. Studies with a score greater than or equal to 8 were classified as high quality, 6–7 as medium quality, and less than 6 as low quality.

The Grading of Recommendations Assessment, Development and Evaluation (GRADE) approach was utilized to assess the certainty of the evidence. This approach employs specific criteria to categorize the quality of evidence into four levels: very low, low, moderate, and high. Two researchers conducted the assessments independently. Any disagreements were then resolved through discussion until they reached a consensus. Observational evidence automatically started at low with the ability to upgrade or downgrade (18).

### 2.4 Statistical analysis

Statistical analysis was performed with RevMan software (Version 5.4, The Cochrane Collaboration). For dichotomous outcomes, the random-effects model was used to calculate the odds ratio (OR) and 95% confidence intervals (CIs). For continuous outcomes, the random-effects model was used to calculate the weighted mean difference (WMD) with 95% CI. *P*-values less than 0.05 was defined as statistically significant. Heterogeneity between studies was assessed by the Cochran Q statistic (chi-square test) and the *I^2^* statistic. As a guide, *P*-value greater than 0.10 indicated no significant heterogeneity. *I^2^* values less than 25% indicated low heterogeneity, ranges from 25% to 50% indicated moderate heterogeneity, and values greater than 50% indicated high heterogeneity (19). The potential for publication bias was evaluated by funnel plots. Subgroup analyses and sensitivity analysis were carried out based on the usage method of PPCDs.

## 3 Results

### 3.1 Study selection and characteristics

**Figure 1:**
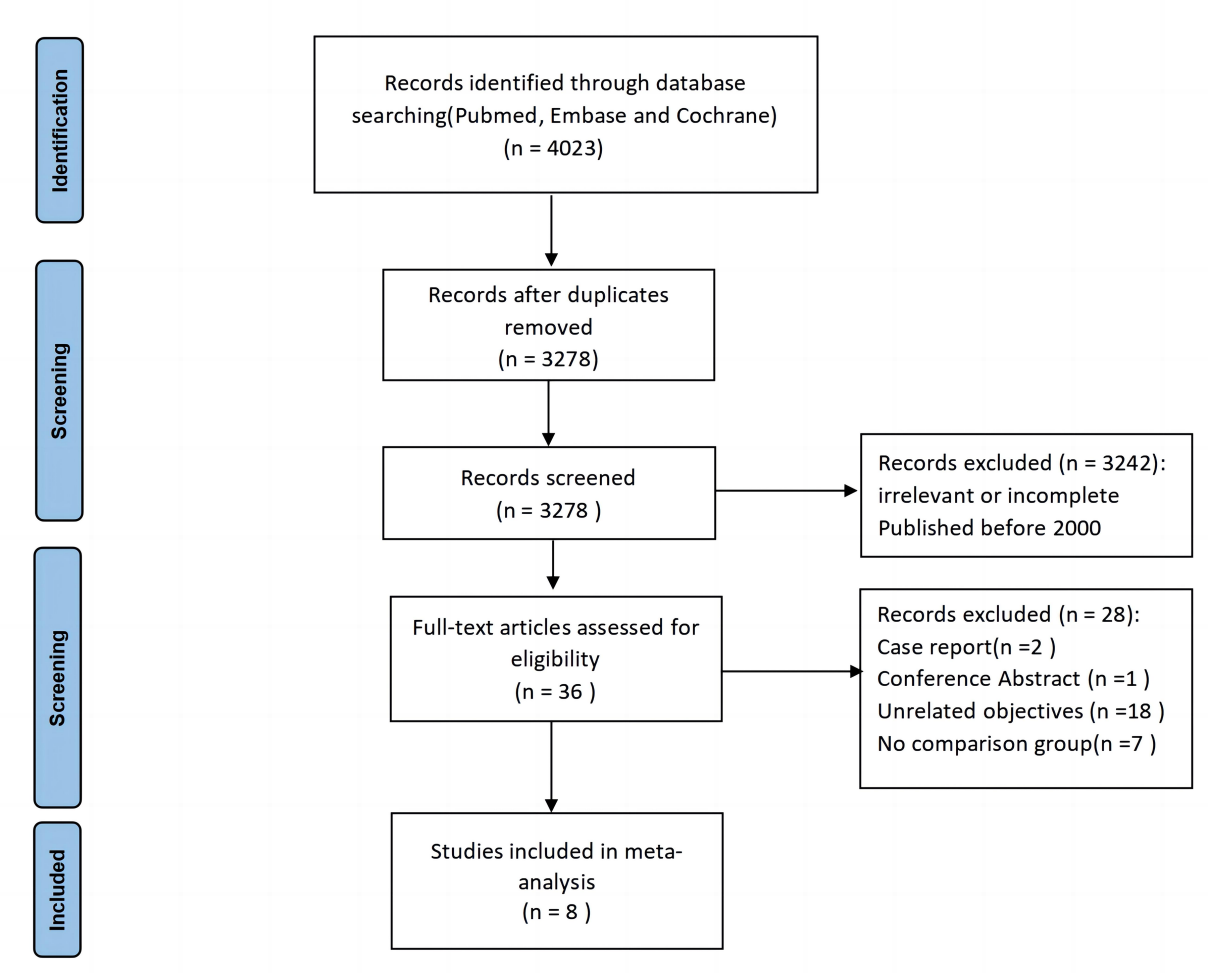
PRISMA flow diagram of literatures screening in the study.

Initially, 4,023 studies were identified through keyword searches. After excluding duplicates, 3,278 studies remained, of which 3,242 were excluded after further review of the titles and abstracts. The remaining 36 were evaluated for eligibility and eight trials were included in both qualitative analysis and quantitative analysis (Figure 1). The main characteristics of the included studies are reported in Table 1. A total of eight retrospective observational studies that compared PPCD with SR for VA-ECMO decannulation were included (13–16, 20–23). The studies included were all single-center studies published between 2016 and 2024. In total, there were 618 patients, with 337 undergoing percutaneous decannulation with PPCDs and 281 undergoing surgical decannulation of VA-ECMO. Among the included studies, two exclusively employed the pre-closure technique in the percutaneous decannulation group (16, 21), five exclusively utilized the post-closure technique (13–15, 20, 23), and one employed both techniques (22). A study reporting on the use of PPCDs for decannulation in VA-ECMO and catheter-type heart pumps did not provide some detailed data (such as gender and age) for the VA-ECMO group (13). The quality assessment revealed that the eight included studies were deemed of high quality according to the NOS standard (Table 2).

**Table 1:** Main characteristics of included studies.

First author	Publication year	Country	Study design	Participants, *n*		Mean Age, years		Male, *n*		Mean BMI, kg/m2		SOFA score		Usage method of PPCDs	Indications for VA-ECMO
				PPCDs	SR	PPCDs	SR	PPCDs	SR	PPCDs	SR	PPCDs	SR		
Majunke	2016	Germany	Retrospective	15	20	61	60	11	16	25.8	27.3	NA	NA	Post-closure	Refractory cardiogenic shock
Hwang	2016	Korea	Retrospective	56	59	53.34	49.76	19	24	23.41	22.93	NA	NA	Post-closure	Refractory cardiogenic shock, refractory cardiac arrest
Pellenc	2020	France	Retrospective	106	48	53.5	53.0	75	34	23.9	24.2	NA	NA	Pre-closure	Patients undergoing lung transplantation due to preoperative respiratory failure secondary to interstitial lung disease associated with pulmonary hypertension, lung transplant patients experiencing severe pulmonary hypertension, right ventricular failure, and heart-constraining surgical maneuvers at any time during surgery
Chandel	2021	USA	Retrospective	51	48	56	58	33	37	27.1	28.4	NA	NA	Pre-closure	Refractory cardiogenic shock
Sun	2023	China	Retrospective	30	11	65.5	52	22	5	28.8	23.1	NA	NA	Pre-closure/Post-closure	Refractory cardiogenic shock, cardiac arrest, or escort of complex interventions
Unoki	2023	Japan	Retrospective	32	36	NA	NA	NA	NA	NA	NA	NA	NA	Post-closure	Severe cardiogenic shock
Roberts	2024	USA	Retrospective	21	27	52	53	18	18	30.0	28.7	NA	NA	Post-closure	Acute myocardial infarction, postcardiotomy cardiogenic shock, cardiac arrest, and refractory cardiogenic shock
Li	2024	China	Retrospective	26	32	47.80	52.90	13	22	NA	NA	7.38	7.69	Post-closure	Refractory cardiogenic shock, refractory cardiac arrest

Data presented as *n* or mean.

PPCDs, Perclose ProGlide closure devices; SR, surgical removal; VA-ECMO, veno-arterial extracorporeal membrane oxygenation; BMI, body mass index; USA, United States of America; NA, not available.

**Table 2:** Quality assessment of the included cohort studies according to the NOS.

Study	Selection				Comparability	Outcome		
	Representativeness of the exposed cohort	Selection of the non-exposed cohort	Ascertainment of exposure	Demonstration that outcome of interest was not present at start of study	Comparability of cohorts on the basis of the design or analysis	Assessment of outcome	Was follow-up long enough for outcomes to occur	Adequacy of follow up of cohorts
Majunke et al. (16)	★	★	★	★	★	★	★	★
Hwang et al. (14)	★	★	★	★	★	★	★	★
Pellenc et al. (16)	★	★	★	★	★	★	★	★
Chandel et al. (21)	★	★	★	★	★	★	★	★
Sun et al. (22)	★	★	★	★	★	★	N/A	★
Unoki et al. (14)	★	★	★	★	★	★	★	★
Roberts et al. (15)	★	★	★	★	★	N/A	★	★
Li et al. (23)	★	★	★	★	★	★	★	★

This table identifies “high” quality choices with a “star”. A study can be awarded a maximum of 1 star for each numbered item within the Selection and Exposure categories. A maximum of 2 stars can be given for Comparability.

★, yes; N/A, Not applicable; NOS, Newcastle-Ottawa scale.

### 3.2 Technical success

**Figure 2:**
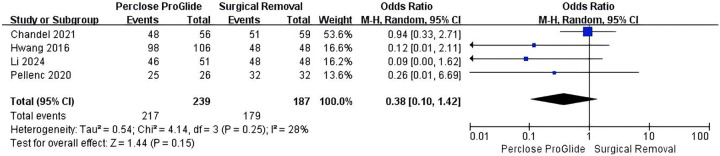
Forest plots of pooled studies comparing technical success between PPCDs and SR for VA-ECMO decannulation.

Data on technical success were provided in six out of the eight included studies (13, 14, 16, 20, 21, 23). In addition, Unoki et al. and Majunke et al. reported that all patients achieved technical success (13, 20). Data from 426 patients could ultimately be utilized for analysis of technical success. Technical success occurred in 90.8% (217 of 239) in the PPCD group and 95.7% (179 of 187) in the SR group. There was no significant difference in technical success rate between the two groups (OR = 0.38, 95% CI 0.10–1.42, *P* = 0.15; Figure 2), with moderate but not significant heterogeneity (*I^2^* = 28%, *P* = 0.25).

### 3.3 Infections at the decannulation site

**Figure 3:**
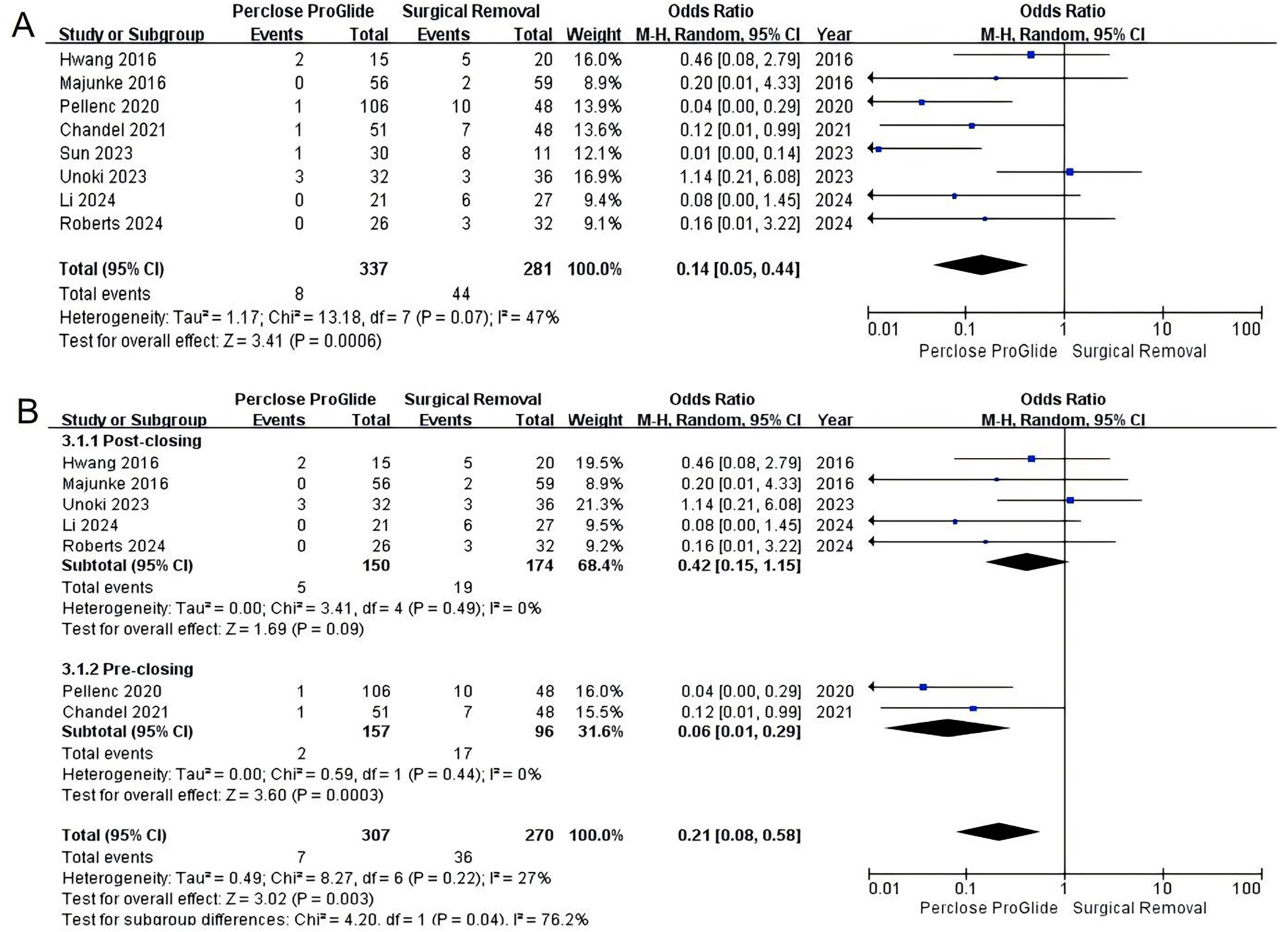
Forest plots of pooled studies comparing infections at the decannulation site between PPCDs and SR for VA-ECMO decannulation. **(A)** OR for infections at the decannulation site; **(B)** Subgroup analysis of OR for infections at the decannulation site.

All studies including 618 patients assessed infections at the decannulation site which occurred in 2.4% (8 of 337) in the PPCD group and 15.7% (44 of 281) in the SR group. A significant reduction in infections at the decannulation site was found in PPCDs vs. SR (OR = 0.14, 95% CI 0.05 to 0.44, *P* = 0.0006; Figure 3A). There was significant moderate heterogeneity across the trials (*I^2^* = 47%, *P* = 0.07). After excluding the study by Sun et al. involving two techniques (22), we conducted a subgroup analysis based on the usage method of PPCDs. The results of subgroup analysis revealed that there was no statistical difference in the incidence of infections at the decannulation site between the percutaneous post-closure group and the SR group (OR = 0.42, 95% CI 0.15–1.15, *P* = 0.09, *I^2^* = 0%; Figure 3B). On the other hand, percutaneous pre-closure technique reduced the risk of infections at the decannulation site compared to SR with significant difference (OR = 0.06, 95% CI 0.01–0.29, *P* = 0.0003, *I^2^* = 0%; Figure 3B).

### 3.4 Bleeding events

**Figure 4:**
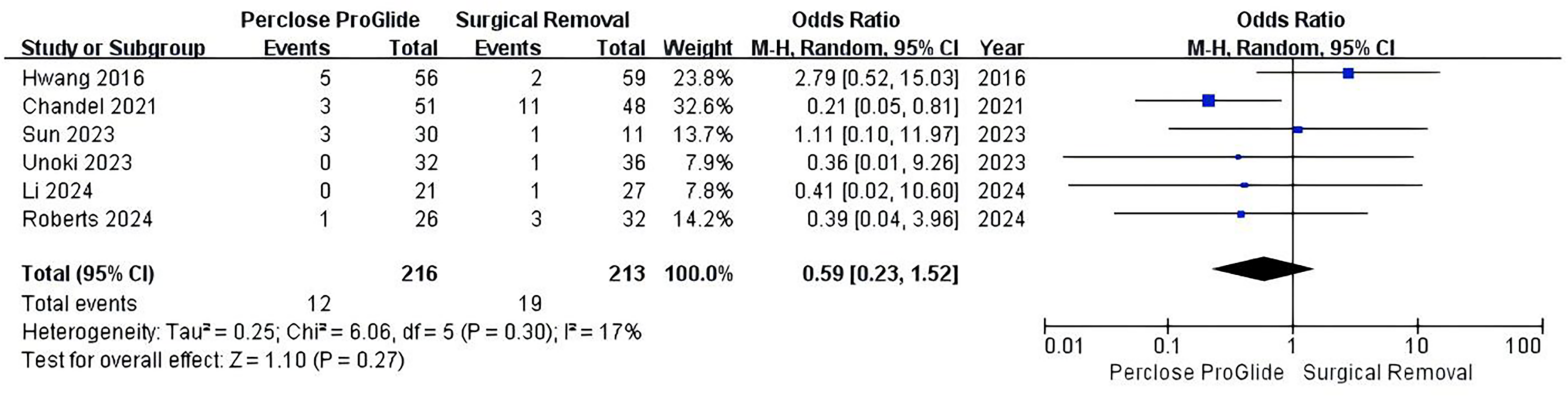
Forest plots of pooled studies comparing bleeding events between PPCDs and SR for VA-ECMO decannulation.

Six studies including 429 patients provided data on bleeding events for the meta-analysis (13–15, 21–23). Bleeding events occurred in 5.6% (12 of 216) in the PPCD group and 8.9% (19 of 213) in the SR group. However, the difference was not statistically significant (OR = 0.59, 95% CI 0.23–1.52, *P* = 0.27; Figure 4). There was mild heterogeneity across the studies (*I^2^* = 17%, *P* = 0.30).

### 3.5 Vascular complications

**Figure 5:**
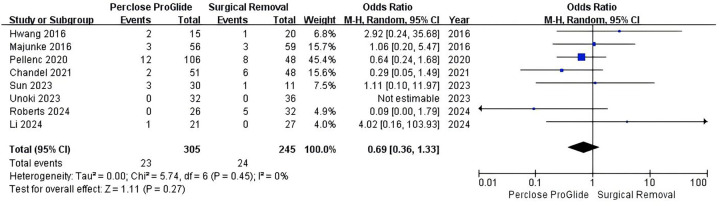
Forest plots of pooled studies comparing vascular complications between PPCDs and SR for VA-ECMO decannulation.

All studies provided data on vascular complications. In the study by Unoki et al., there were no vascular complications in either group (13). In the other seven studies, the incidence of vascular complications was 7.5% (23 of 305) in the PPCD group and 9.8% (24 of 245) in the SR group and the result indicated that the two groups have a comparable risk of vascular complications (OR = 0.69, 95% CI 0.36–1.33, *P* = 0.27; Figure 5), without heterogeneity (*I^2^* = 0%, *P* = 0.45).

### 3.6 Overall complications

**Figure 6:**
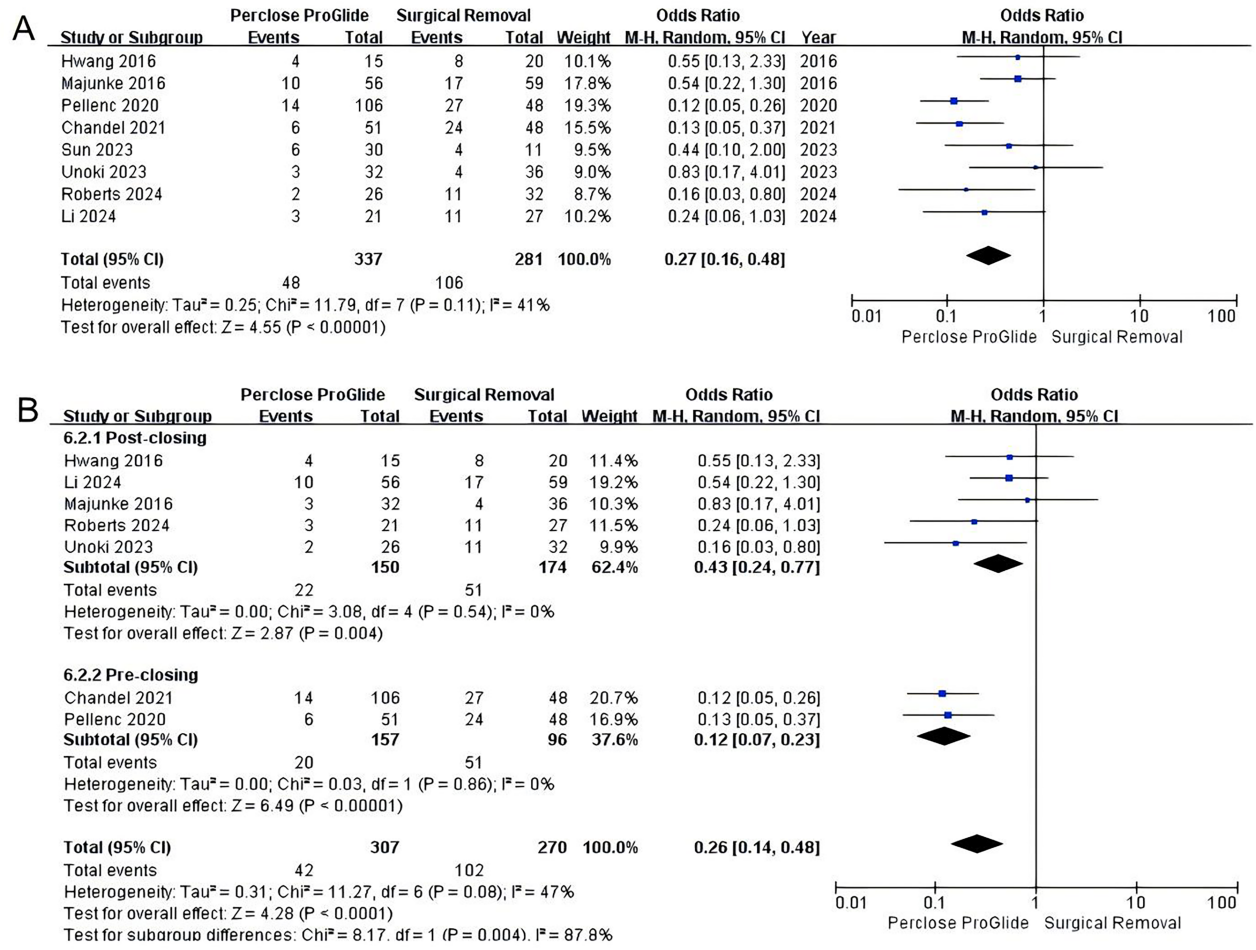
Forest plots of pooled studies comparing overall complications between PPCDs and SR for VA-ECMO decannulation. **(A)** OR for overall complications; **(B)** Subgroup analysis of OR for overall complications.

All studies provided data on overall complications. There was a positive impact for patients undergoing PPCDs in terms of reducing overall complications (OR = 0.27, 95% CI 0.16–0.48, *P* < 0.00001; Figure 6A). The between-study heterogeneity was statistically significant in this analysis (*I^2^* = 41%, *P* = 0.11). To address the heterogeneity, we conducted a subgroup analysis based on the usage method of PPCDs and also excluded the study by Sun et al. from our evaluation. The results of the subgroup analysis revealed that both the pre-closure and post-closure techniques significantly reduced overall complications compared to SR (OR = 0.12, 95% CI 0.07–0.23, *P* < 0.00001, *I^2^* = 0%; Figure 6B), (OR = 0.43, 95% CI 0.24–0.77, *P* = 0.004, *I^2^* = 0%; Figure 6B).

### 3.7 Mortality

**Figure 7:**
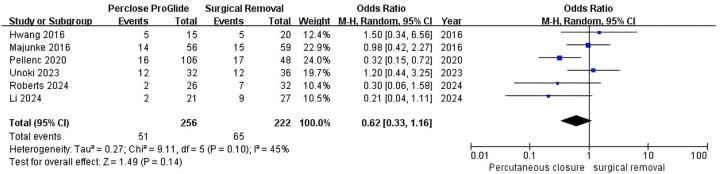
Forest plots of pooled studies comparing mortality between PPCDs and SR for VA-ECMO decannulation.

Six studies reported data on mortality (13–16, 20, 23). The pooled results indicated no statistical difference between the PPCD group and the SR group (OR = 0.62, 95% CI 0.33–1.16, *P* = 0.14; Figure 7), with significant moderate heterogeneity (*I^2^* = 45%, *P* = 0.10). Subgroup analysis based on the usage method of PPCDs could not identify the source of heterogeneity.

### 3.8 Duration of hospitalisation

**Figure 8:**
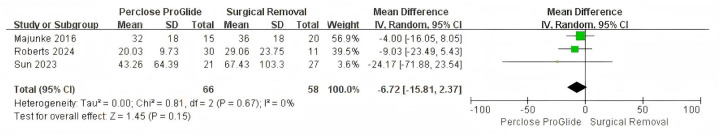
Forest plots of pooled studies comparing duration of hospitalisation between PPCDs and SR for VA-ECMO decannulation.

Out of the eight studies included, three provided information on the duration of hospitalization (15, 20, 22). The results indicated that there was no statistically significant difference in the duration of hospitalisation between the PPCD group and the SR group (WMD = −6.72, 95% CI −15.81 to 2.37, *P* = 0.15, *I^2^* = 0%; Figure 8).

### 3.9 Sensitivity analysis

The study by Sun et al. was the only one that included two techniques for the use of PPCDs amongst the included studies (22). However, the results of two different closure methods that were not reported separately might result in low quality of data. Therefore, a sensitivity analysis was performed by excluding the study conducted by Sun et al. The removal did not change the direction of all results. In reducing the incidence of infections at the removal site and overall complications, PPCDs still demonstrated a clear superiority over SR (OR = 0.21, 95% CI 0.08–0.58, *P* = 0.003; *I^2^* = 27%) (OR = 0.26, 95% CI 0.14–0.48, *P* < 0.001, *I^2^* = 47%). In the revised analysis, there was no difference of bleeding events and vascular complications in odds for PPCDs against SR and *p*-value was still not significant (OR = 0.54, 95% CI 0.17–1.66, *P* = 0.28, *I^2^* = 30%) (OR = 0.67, 95% CI 0.32–1.43, *P* = 0.30, *I^2^* = 10%).

### 3.10 Publication bias

However, due to the limited number of articles included in this meta-analysis (fewer than 10), we couldn't assess publication bias.

### 3.11 GRADE assessment

We evaluated the quality of evidence for primary and secondary outcomes (Supplementary Table S2). Given that all the studies included were retrospective, the evidence for technical success, catheter site infection, bleeding events, vascular complications, overall complications and duration of hospitalisation was considered to be of low quality. The evidence relating to mortality was downgraded to very low quality due to serious limitations of inconsistency.

## 4 Discussion

Patients undergoing VA-ECMO are frequently in critical condition. Meticulous attention to and enhancement of management details at every step of VA-ECMO, from cannulation to utilization and decannulation, can significantly improve patient outcomes. At present, a unified consensus or recommendation regarding the selection of decannulation methods for VA-ECMO has not been established. Although the utilization of PPCDs, which leverage suture-based technology, is emerging as an increasingly favored approach in the decannulation process of VA-ECMO, there remains a paucity of robust clinical evidence to definitively prioritize their use over traditional SR. This meta-analysis, encompassing data from eight retrospective cohort studies, demonstrated that the technical success rate of PPCDs was equivalent to that achieved with SR. Regarding safety profiles, no significant disparities were observed between PPCDs and SR in the incidence of vascular complications, bleeding events, mortality rates and duration of hospitalisation. Importantly, the use of PPCDs in VA-ECMO decannulation, particularly the pre-closure technique, was associated with a significant reduction in the risk of infections at the decannulation site and a decreased incidence of overall complications. Consequently, under appropriate clinical conditions, the pre-closure technique utilizing PPCDs should be prioritized as the primary option for VA-ECMO decannulation.

Zhu et al.'s meta-analysis conducted a comparison between two distinct percutaneous vascular closure devices and SR for VA-ECMO decannulation (24). The study demonstrated that the use of percutaneous closure devices was associated with a reduced rate of overall complications and infections at the decannulation site, findings that are consistent with the outcomes of our research. Additionally, their study demonstrated a significant reduction in bleeding events associated with the use of vascular closure devices. However, our analysis, which included data from six studies, did not confirm a similar advantage for the PPCD in reducing bleeding events. Au et al. and Scherer et al. have reported that, when compared with the use of the PPCD, the MCD was associated with a significantly reduced incidence of severe bleeding events during the process of VA-ECMO decannulation (25, 26). The divergence observed between the findings of Zhu et al. and our study may be attributed to the fact that, in their meta-analysis, the percutaneous vascular closure devices utilized in two of the included studies were the plug-based MCD.

Furthermore, in comparison with the study by Zhu et al., our analysis included two additional studies that employed the PPCDs and we conducted a comparative evaluation of the technical success rates between PPCDs and SR (13, 15). We observed that the technical success rates of PPCDs were comparable to those of SR. This result appears to mitigate concerns regarding the potential lower success rate of PPCDs due to the intricacies of their deployment process. However, the foundation of technical success lies in a profound familiarity with the preparatory work, procedural steps, and technical application of percutaneous closure devices. Given the limited number of cases and the steep learning curve, training on the use of PPCDs can be carried out through simulated scenarios (10).

Our results demonstrated that the use of PPCDs compared to SR reduced infections at the decannulation site. This significant reduction may be associated with two key factors. Firstly, unlike SR, the design of PPCDs enables closure with minimal tissue contact, thereby reducing tissue damage, inflammatory responses, and avoiding direct exposure of deep tissues, which in turn lowers the risk of infection. Secondly, the simplicity of PPCD operation allows for rapid and effective sealing of vascular puncture sites, reducing blood leakage and consequently decreasing the risk of infection. Additionally, regarding infections at the decannulation site and overall complications, considering the significant heterogeneity of the results, we conducted a subgroup analysis based on the usage method of PPCDs. In the subgroup analysis pertaining to decannulation site infections, we observed no significant heterogeneity within any subgroups, suggesting that the method of PPCD usage was a significant source of heterogeneity. Previous perspectives have raised concerns that the use of pre-closure techniques to pre-embed sutures might increase the incidence of infection, given the uncertain duration of continuous VA-ECMO support (23). However, the findings from our subgroup analysis indicated that the pre-closure technique offered significant benefits in reducing infections at the decannulation site. In contrast, the post-closure technique did not demonstrate a significant advantage over traditional surgical approaches. These results challenge our initial assumptions and suggest a preference for employing pre-closure technology in clinical practice. Concurrently, pre-closure technology is the most frequently reported vascular repair strategy in complex endovascular procedures, such as transcatheter aortic valve implantation or endovascular aortic repair, and has been proven to be a straightforward, secure, and efficacious approach to arterial repair (27, 28). However, VA-ECMO is often implemented in emergency scenarios where the requisite time, equipment, and team support for pre-closure may not be readily accessible. Furthermore, the additional time required to deploy pre-closure devices before ECMO support may adversely affect the condition of patients in emergency situations. To date, no retrospective or randomized controlled trials have been conducted to directly compare the pre-closure and post-closure techniques using PPCDs for VA-ECMO decannulation. In terms of overall complication results, the subgroup analysis showed that both pre-closure and post-closure techniques were superior to surgical procedures.

Patients who underwent VA-ECMO decannulation via PPCDs or SR demonstrated no significant differences in mortality rates. Notwithstanding the significant heterogeneity observed in the outcomes, this heterogeneity was not found to be related to the method of PPCD usage. Given the unique clinical profiles of patients receiving VA-ECMO, the variability in disease presentation, complexity, and severity may be more reflective of the sources of heterogeneity observed in our study (29–31).

In this study, we have noted that among the eight included studies, only one mentioned the use of antibiotics (22), and three discussed the anticoagulation protocols (13, 16, 22). This highlights a significant deficiency in data regarding infection prevention and anticoagulation management. Anticoagulation therapy is of paramount importance for VA-ECMO patients and is also associated with bleeding events. Rational use of antibiotics can help reduce the risk of infection. These factors may significantly impact the safety of the two decannulation methods. However, due to the lack of sufficient data, we are unable to accurately assess the role of these factors in the comparison of the two decannulation methods, making it difficult to comprehensively judge the differences in safety between the two methods. Furthermore, although six studies mentioned the importance of institutional experience with vascular closure devices or VA-ECMO, none provided further elaboration (14, 15, 20–23). The lack of detailed descriptions and quantitative analysis of institutional experience prevents us from fully considering the impact of this factor on the study results. The absence or inconsistency of this information limits the generalizability of the study conclusions. In different medical environments, differences in anticoagulation and infection prevention strategies, as well as team experience, may lead to variations in the actual effects of the two decannulation methods. Therefore, when applying the conclusions of this study to clinical practice, it is necessary to carefully consider these potential influencing factors, and the applicability is somewhat uncertain. Future studies should pay more attention to the collection and analysis of these confounding factors to provide more comprehensive and convincing evidence, thereby better guiding clinical practice.

To the best of our knowledge, this represents the only meta-analysis exclusively comparing the use of suture-based PPCDs vs. SR in the application of VA-ECMO decannulation. The findings of this study contribute valuable evidence supporting the utilization of suture-based closure devices for percutaneous VA-ECMO decannulation. For the first time in similar studies, the potential differences between pre-closure and post-closure techniques of PPCDs on VA-ECMO decannulation outcomes were examined and analyzed through subgroup analysis, aiding clinicians in making more informed decisions about VA-ECMO decannulation tailored to their specific clinical contexts. There are several limitations to our study. First, the lack of randomized controlled trials of the two interventions that met our inclusion criteria might lead to potential selection bias. Second, due to the limited number of included studies, it was impossible to create a funnel plot to supplement the evaluation of publication bias. Finally, the literature search was limited to studies published in English.

## 5 Conclusion

In conclusion, our meta-analysis indicates that PPCDs offer a safe and feasible approach to VA-ECMO decannulation. Compared with SR, the use of PPCDs is associated with a significant reduction in overall complications, particularly with respect to the pre-closure technique, which markedly diminishes the incidence of infections at the decannulation site. When conditions permit, the pre-closure technique should be given priority for VA-ECMO decannulation. However, these conclusions necessitate further validation through high-quality, large-sample randomized controlled trials.
