# Supplementary material for: Perclose ProGlide closure devices vs. surgical removal for veno-arterial extracorporeal membrane oxygenation decannulation: a meta-analysis
Source: Front Cardiovasc Med. 2025 Feb 28;12:1482305. doi: 10.3389/fcvm.2025.1482305 (PMC11906672; doi:10.3389/fcvm.2025.1482305)
Supplement: Supplementary file 1 [file Table1.doc]

Supplementary Material

| **Supplementary Table 1 Detailed search strategies.**  **(from database inception to May 1, 2024)** | |
| --- | --- |
| **Databases** | **Search strings** |
| **Pubmed** | (((((((((Extracorporeal membrane oxygenation[Title/Abstract])) OR (ECMO[Title/Abstract])) OR (Extracorporeal life support system[Title/Abstract])) OR (ECLS[Title/Abstract])) OR (Mechanical circulatory support[Title/Abstract])) OR (MCS[Title/Abstract])) OR (Extracorporeal membrane oxygenation[Mesh]) ) AND ((((((Perclose[Title/Abstract])) OR (Proglide[Title/Abstract])) OR (ProStyle[Title/Abstract])) OR (closure[Title/Abstract])) OR (percutaneous[Title/Abstract]) )) AND ((Surgical Procedures, Operative[Mesh]) OR ((((((surgical[Title/Abstract])) OR (surgery[Title/Abstract])) OR (open[Title/Abstract])) OR (repair[Title/Abstract])) OR (operative[Title/Abstract]))) |
| **The Cochrane Library** | (MeSH descriptor: [Extracorporeal Membrane Oxygenation] explode all trees OR (extracorporeal membrane oxygenation):ti,ab,kw OR (ECMO):ti,ab,kw OR (Extracorporeal life support system):ti,ab,kw OR (ECLS):ti,ab,kw OR (Mechanical circulatory support):ti,ab,kw OR (MCS):ti,ab,kw) AND ((Perclose):ti,ab,kw OR (Proglide):ti,ab,kw OR (ProStyle):ti,ab,kw OR (percutaneous):ti,ab,kw OR (closure):ti,ab,kw) AND (MeSH descriptor: [General Surgery] explode all trees OR (surgical):ti,ab,kw OR (surgery):ti,ab,kw OR (open):ti,ab,kw OR (repair):ti,ab,kw OR (operative):ti,ab,kw) |
| **EMBASE** | ('extracorporeal oxygenation'/exp OR 'extracorporeal membrane oxygenation':ab,ti OR 'ecmo':ab,ti OR 'extracorporeal life support system':ab,ti OR 'ecls':ab,ti OR 'mechanical circulatory support':ab,ti OR 'mcs':ab,ti) AND ('perclose':ab,ti OR 'proglide':ab,ti OR 'prostyle':ab,ti OR 'percutaneous':ab,ti OR 'closure':ab,ti) AND ('surgery':ab,ti OR 'surgical':ab,ti OR 'open':ab,ti OR 'repair':ab,ti OR 'operative':ab,ti) |

| **Supplementary Table 2 GRADE Assessment of meta-analytic results.** | | | | | | | | | |
| --- | --- | --- | --- | --- | --- | --- | --- | --- | --- |
| Outcome | No of studies | Design | Risk of bias | Inconsistency | Indirectness | Imprecision | Publication bias | Other considerations | Quality of evidence |
| Technical success | 6 | observational studies | no serious | no serious | no serious | no serious | no serious | None | Low |
| Infections at the decannulation site | 8 | observational studies | no serious | no serious | no serious | no serious | no serious | None | Low |
| Bleeding events | 6 | observational studies | no serious | no serious | no serious | no serious | no serious | None | Low |
| Vascular complications | 8 | observational studies | no serious | no serious | no serious | no serious | no serious | None | Low |
| Overall  complications | 8 | observational studies | no serious | no serious | no serious | no serious | no serious | None | Low |
| Mortality | 6 | observational studies | no serious | Serious ^a^ | no serious | no serious | no serious | None | Very low |
| Duration of hospitalisation | 3 | observational studies | no serious | no serious | no serious | no serious | no serious | None | Low |
| The quality of evidence is divided into 4 levels using GRADE system (high, moderate, low, very low).  ^a^ Downgraded if there was a substantial unexplained heterogeneity (*I*^2^ > 45%, *P* < 0.10) that was unexplained by subgroup analyses or other means. | | | | | | | | | |
